# SangeR: the high-throughput Sanger sequencing analysis pipeline

**DOI:** 10.1093/bioadv/vbac009

**Published:** 2022-01-31

**Authors:** Kai Schmid, Hildegard Dohmen, Nadja Ritschel, Carmen Selignow, Jochen Zohner, Jannik Sehring, Till Acker, Daniel Amsel

**Affiliations:** 1 Department of Neuropathology, Justus-Liebig University Giessen, Arndtstr, 16 D-35392 Giessen, Germany; 2 Department of Medical Informatics, Justus-Liebig University Giessen, Rudolf-Buchheim-Str, 6 D-35396 Giessen, Germany

## Abstract

**Summary:**

In the era of next generation sequencing and beyond, the Sanger technique is still widely used for variant verification of inconclusive or ambiguous high-throughput sequencing results or as a low-cost molecular genetical analysis tool for single targets in many fields of study. Many analysis steps need time-consuming manual intervention. Therefore, we present here a pipeline-capable high-throughput solution with an optional Shiny web interface, that provides a binary mutation decision of hotspots together with plotted chromatograms including annotations via flat files.

**Availability and implementation:**

SangeR is freely available at https://github.com/Neuropathology-Giessen/SangeR and https://hub.docker.com/repository/docker/kaischmid/sange_r

**Contact:**

Kai.Schmid@patho.med.uni-giessen.de or Daniel.Amsel@patho.med.uni-giessen.de

**Supplementary information:**

Supplementary data are available at *Bioinformatics* online.

## 1 Introduction

High-throughput sequencing techniques have become established in routine research in the past years, not only because of the decreasing costs per sample but also because of their high-throughput and generation of high-quality data. However, these techniques also have weaknesses. Thus, a next generation sequencing (NGS) approach can go beyond the scope of a study in terms of time and money if, e.g. the presence of a point mutation in a specific region of a single gene is to be investigated. In exome or targeted sequencing, it is also possible that the fragments obtained may be too small, resulting in a lower coverage of regions than expected (Nielsen *et al.*, 2021). This may then lead to results that either cannot clearly be interpreted or cannot be analyzed at all. These regions must then be reanalyzed. Finally, Sanger sequencing is frequently used to verify mutations identified by NGS. For these and other cases, Sanger sequencing remains a grateful option, as it is relatively inexpensive and delivers results relatively quickly.

There are many software tools for the analysis of Sanger raw files. Some of them are free, such as GLASS (Karol *et al.*, 2017), SeqTrace (Stucky, 2012) or BioEdit (Hall, 1999) and some are licensed products with payment options, such as Geneious (Prime, 2019) and CLCbio (QIAGEN, 2021).

What they all have in common, however, is the approach of explorative and visual sequence analysis. This means that the sequenced file is loaded and can be checked for any kind of aberration in a graphical user interface (GUI). The application of such a GUI takes time e.g. for the preparation of the reference sequences, the visual inspection of the sequence or for the export of the results, with each sequence being treated one by one.

It is our understanding, that in most cases inconclusive samples are sequenced with the Sanger technique, because the regions of interest are already well-characterized, such as somatic or germline hot spot single nucleotide polymorphisms (SNPs) mutations or characteristic gene fusions. So, the researcher looks for a particular codon or region in the sequenced region and checks if it is altered or not.

We present here a streamlined pipeline optimized for high-throughput and database communication to reduce human intervention. One can implement it into a fully automated structure with a listener on a specific input folder, the results are then written to the selected output folder. Also, one can use the implemented Shiny web interface with the upload function and view the results directly in the interface. The results can also be forwarded to a specific folder to archive them.

## 2 Materials and methods

### 2.1 Implementation

The SangeR pipeline uses R, Nextflow (Di Tommaso *et al.*, 2017), Shiny (Chang *et al.*, 2021) as well as a Docker container to ensure ease of use and high-throughput of .ab1 files. References are automatically downloaded from the biomaRt (Durinck *et al.*, 2019; Howe *et al*., 2021) database. By using the data format of .ab1 files, it automatically parses the gene name and the ID assigned to the probe. The read-in can be configured to specify cutoff, offset, or a minimum sequencing length, allowing the user to assign the correct sensitivity for their use case if required. To detect the mutations, the reference for the gene is obtained with the help of biomaRt.

The parameters for the reference can also be fine-tuned by the user to obtain different hosts, datasets, marts or also a specific upstream region to cover specific promoter regions. With all information gathered, SangeR checks all possible orientations of the alignment, selects the best-fitting one and annotates all mutations. The sensitivity and specificity of the mutation detection are determined by the parameters selected in the read-in.

A histogram is generated for each mutated position which can be stored to a database with the corresponding mutation tag. The tag consists of the reference nucleotide or amino acid (if the mutation is in the exon), the chromosome position or position in the amino acid chain and the nucleotide or amino acid change caused by the mutation.

To create a histogram for all points of interest even if no mutation is detected a .csv file can be provided to SangeR. For each of the defined locations, a histogram is created in combination with a tag lacking the nucleotide/amino acid at the last position.

*SangeR package*: The pipeline consists of the published SangeR R-package which can be obtained through the GitHub repository.

*Nextflow pipeline*: To process large quantities of files, we provide SangeR as a Nextflow script. Nextflow is a reactive workflow framework for orchestrating scripts to enable high data throughput. The provided script can be easily configured to observe a specific directory and run the analysis in an automated and scheduled manner.

*Rshiny server*: The usability of SangeR is enhanced by a Shiny server which provides the entire functionality of the pipeline with an easy-to-use graphical web interface.

The entire tool can be easily set up with the scripts provided in the Git repository: https://github.com/Neuropathology-Giessen/SangeR.

## 3 Results

The SangeR tool has been applied to cell line data as a use case example. The positions tested were selected in contexts relevant to brain tumors (see Supplementary Table S1).

The resulting chromatograms visualize the measured intensities for the four channels of the Sanger sequencing. The critical position is displayed in the center of the plot with five flanking nucleotides in both directions. The indicated tag on top of the histogram provides information about the detected mutation (see Supplementary Figs S2, S3 and S4).

The validation and testing of SangeR were carried out in two phases. First, good practice in R-package development requires us to test all functions, which we performed with the package devtools (Wickham *et al.*, 2021). Second, we tested the functionality of the tool against different positions of interest and validated the results against Geneious (see Supplementary Table S1).

## 4 Conclusion

The presented pipeline was implemented with the focus on high-throughput of .ab1 files and positions that are repeatedly analyzed. This focus makes the tool easy to establish in laboratories and studies which require repeated testing of specific SNPs e.g. to verify the identity of cell lines, check for the presence of marker mutations or validate NGS results. Importantly, the pipeline can easily be adapted for the analysis of somatic or germline hot spot SNPs in the context of tumor companion diagnostics for research questions or human genetics analyses. In the future, the pipeline will be used to perform large-scale statistical analysis of archived data. In this version, the tool uses a statistical threshold. In the future development, the use of a machine learning-based algorithm could improve the accuracy and minimize the fine-tuning of the threshold required.

## Supplementary Material

vbac009_Supplementary_Data
