# Supplementary material for: SangeR: the high-throughput Sanger sequencing analysis pipeline
Source: Bioinform Adv. 2022 Jan 31;2(1):vbac009. doi: 10.1093/bioadv/vbac009 (PMC9710597; doi:10.1093/bioadv/vbac009)
Supplement: vbac009_Supplementary_Data [file vbac009_supplementary_data.docx]

**Supplementary Material**


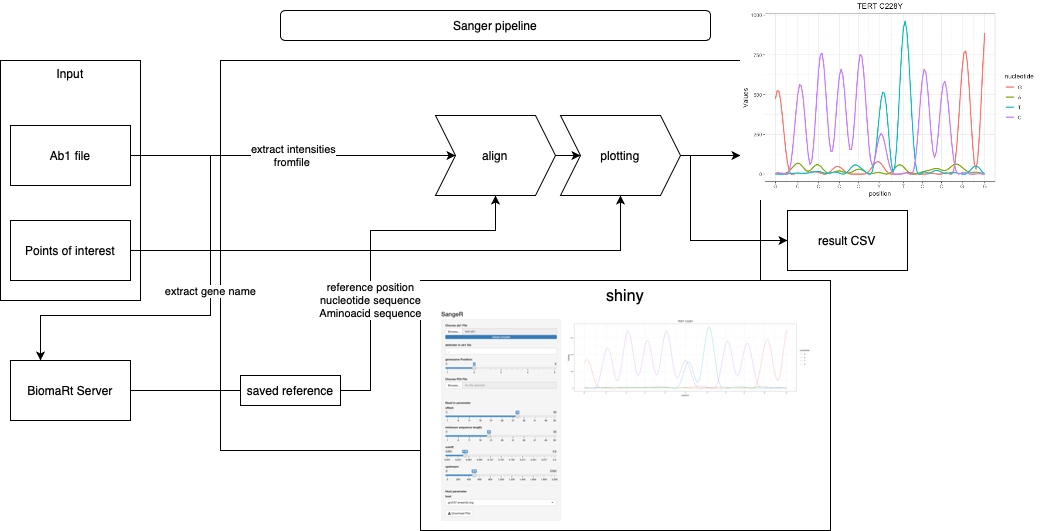


Figure 1: *Flowchart of SangeR functionality. After extracting the gene name, the reference information is loaded from Ensembl* (Kevin L Howe 2021) *or from a locally stored file. This is followed by the alignment to the Sanger sequence.*  *Histograms are printed for mutations found or specific points of interest and a .csv result file is generated. All functionalities can also be accessed through the Shiny user interface. In addition, the complete workflow can be automated by using the provided nextflow pipeline.*

Table 1: Summary of the different established human cell lines and patient samples used in this study with the corresponding investigated point mutations of the TERT promoter, or the genes IDH1, IDH2, and H3F3A.

|  | | **TERT promoter** | | **IDH1** | **IDH1** | **IDH2** | **H3F3A** | |
| --- | --- | --- | --- | --- | --- | --- | --- | --- |
| **#** | **Sample/Cell line** | **C250** | **C228** | **R132** | **G105** | **R172** | **K28** | **G34** |
| 1 | G55 | **C250T** | WT | WT | WT | WT | WT | WT |
| 2 | G141 | WT | **C228T** | WT | WT | WT | WT | WT |
| 3 | HGBM | WT | **C228T** | WT | WT | WT | WT | WT |
| 4 | U-87-MG | WT | **C228T** | WT | WT | WT | WT | WT |
| 5 | U-118-MG | WT | **C228T** | WT | WT | WT | WT | WT |
| 6 | LN-229 | WT | **C228Y** | WT | WT | WT | WT | WT |
| 7 | GBM46x | WT | **C228T** | WT | WT | WT | WT | WT |
| 8 | Patient 1 | WT | WT | **R132H** | **G105G** | WT | WT | WT |
| 9 | Patient 2 | WT | WT | **R132H** | WT | WT | WT | WT |
| 10 | Patient 3 | WT | WT | WT | WT | **R172M** | WT | WT |
| 11 | Patient 4 | WT | WT | WT | WT | WT | **R028M** | WT |
| 12 | Patient 5 | WT | WT | WT | WT | WT | WT | **G34R** |

Figure 2: Chromatograms of the respective cell line, showing the detected TERT promoter mutations.

| 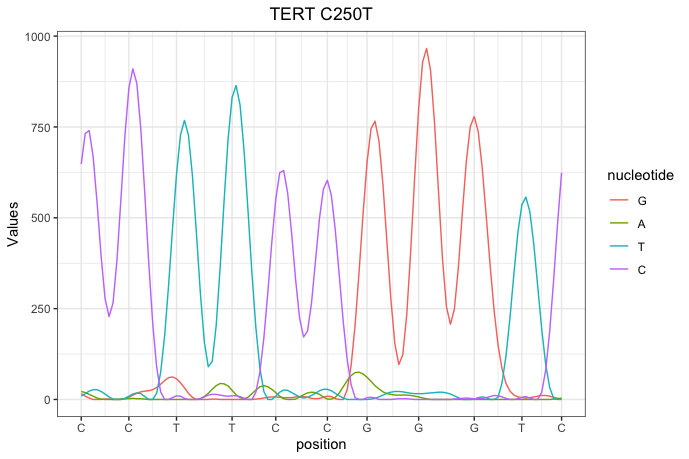 | 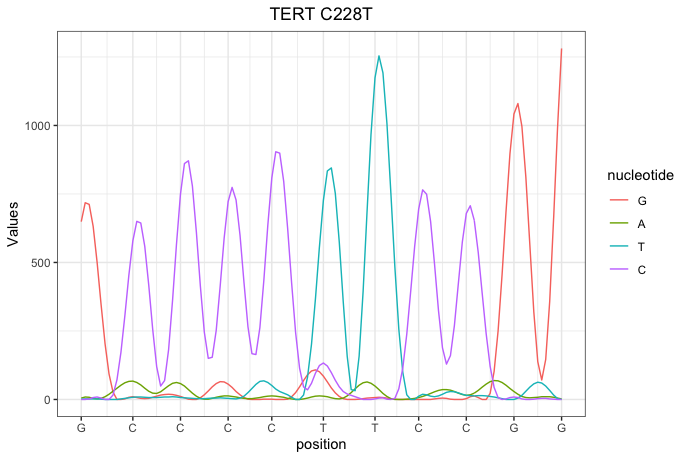 |
| --- | --- |
| G55 | G141 |
| 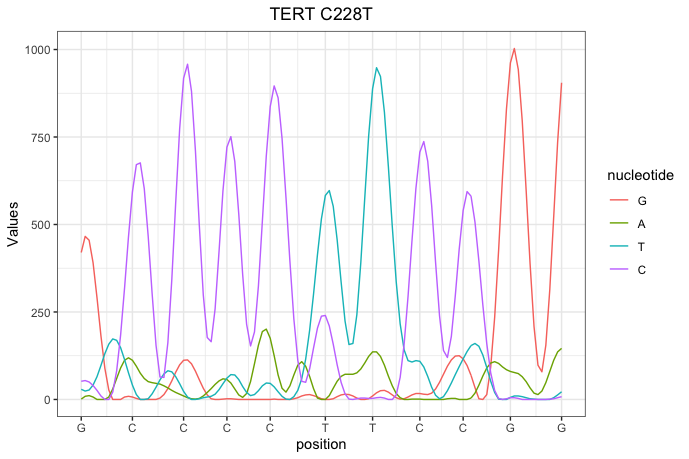 | 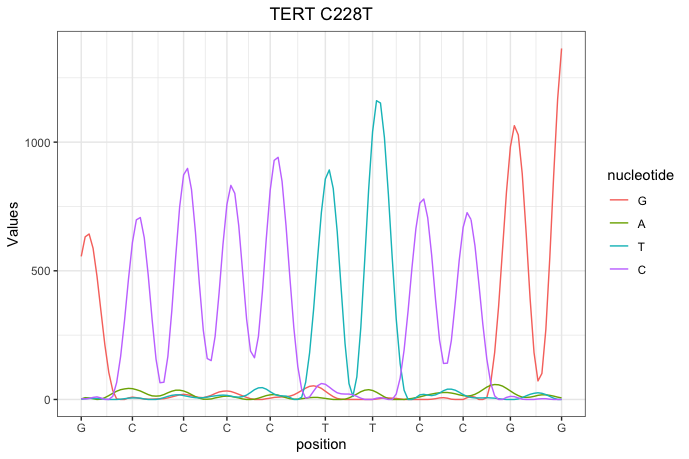 |
| HGBM | U-87 |

Figure 3: table with the chromatograms of each cell line.

| 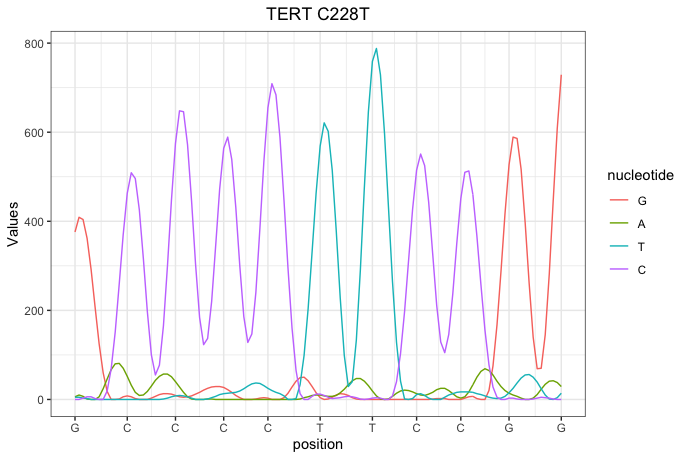 | 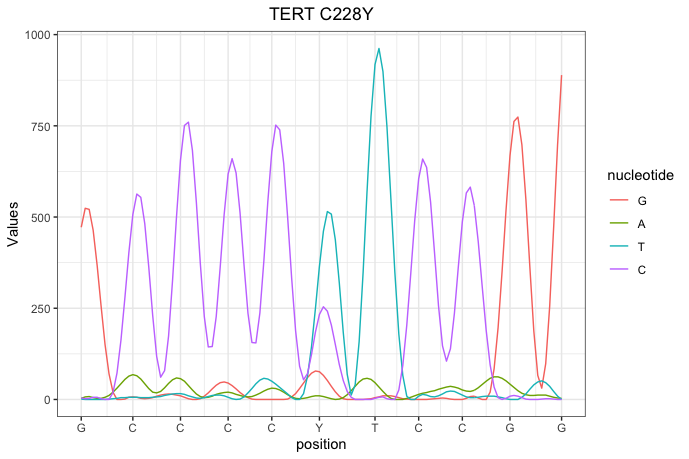 |
| --- | --- |
| **HGBM** | **LN 229** |
| **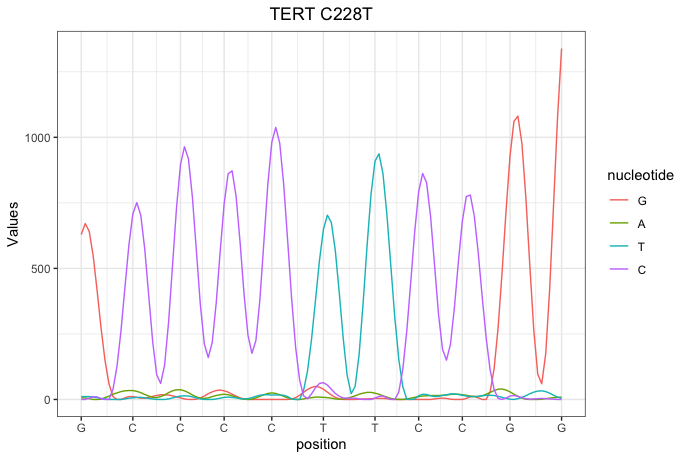** |  |
| **GBM46x** |  |

Figure 4: Chromatograms of the sequenced DNA of patient 1 and 2, showing the foundIDH1 mutations.

| 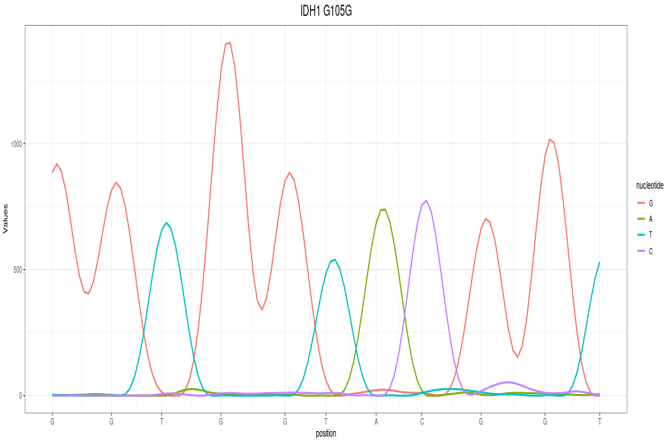 | 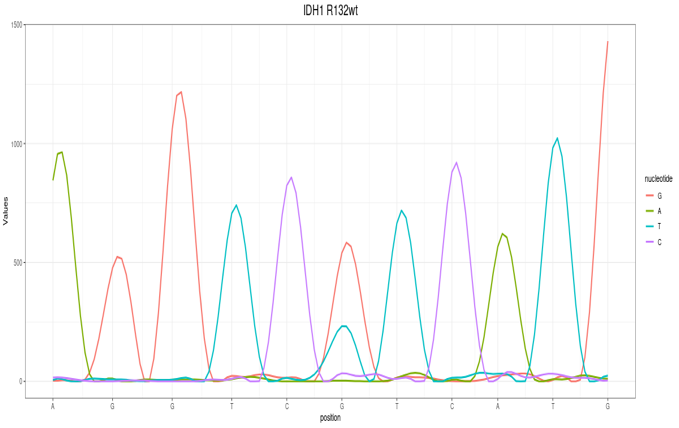 |
| --- | --- |
| Silent mutation G105G from patient 1 | Not recognized R132L mutation from patient 1 (found by preset of POI) |
| 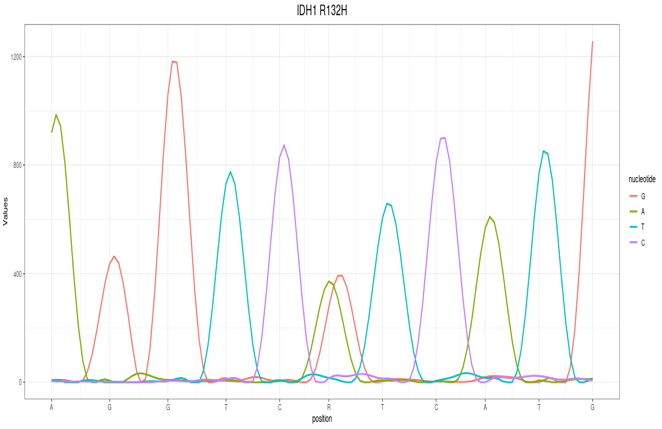 | 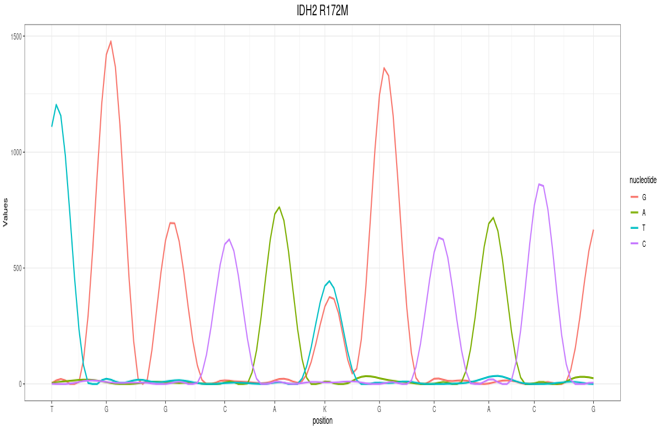 |
| R132H mutation from patient 2 | R172M mutation from patient 3 |
| 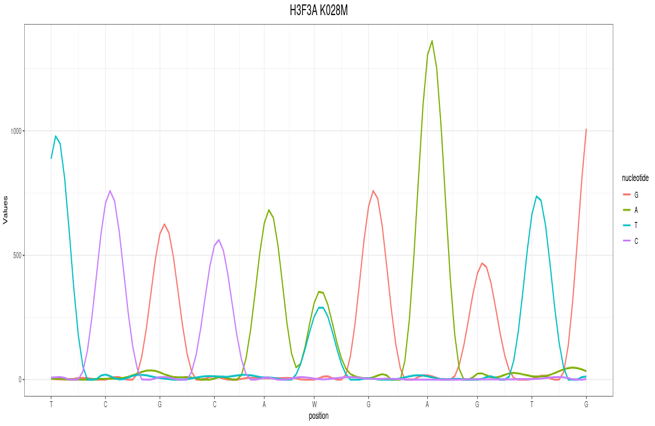 | 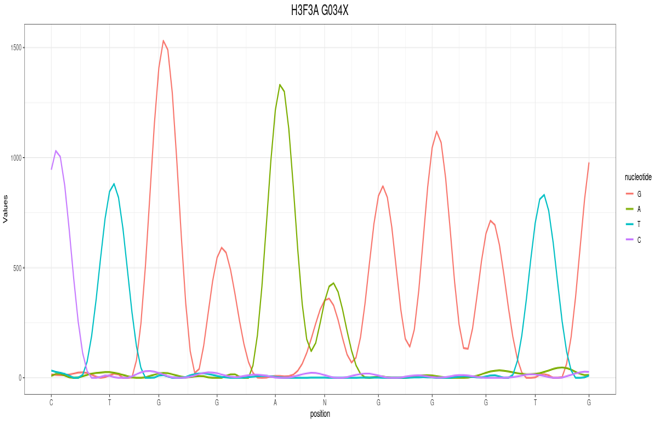 |
| K028M mutation from patient 4 | G034R mutation from patient 5 |
